# Contrast Medium Use in Computed Tomography for Patients Presenting with Headache: 4-year Retrospective Two-Center Study in Central and Western Regions of Ghana

**DOI:** 10.1155/2022/4736455

**Published:** 2022-10-04

**Authors:** Bashiru Babatunde Jimah, Benjamin Dabo Sarkodie, Asare Kwaku Offei, Ewurama Andam Idun, Dorothea Anim, Edmund Brakohiapa, Benard Ohene Botwe

**Affiliations:** ^1^University of Cape Coast, School of Medical Science, Department of Medical Imaging, Cape Coast, Ghana; ^2^University of Ghana School of Medical and Dental Science, Department of Radiology, Accra, Ghana; ^3^Korle Bu Teaching Hospital, Department of Surgery, Accra, Ghana; ^4^37 Military Hospital, Department of Radiology, Accra, Ghana; ^5^Korle Bu Teaching Hospital, Department of Radiology, Accra, Ghana; ^6^Department of Radiography, School of Biomedical & Allied Health Sciences, University of Ghana, Accra, Ghana

## Abstract

**Background:**

Contrast medium (CM) administration during computed tomography (CT) enhances the accuracy in the detection and interpretation of abnormalities. Evidence from literature also validate the essence of CM in imaging studies. CT, by virtue of its ubiquity, ease of use, speed, and lower financial footprint, is usually the first investigation in cases of headache. Through a multicenter retrospective analysis, we compared findings of contrast-enhanced CT (CECT) to noncontrast-enhanced CT (NCECT) head examinations among patients presenting with headache.

**Methods:**

A multicenter retrospective analysis of four years' CT head examination data at two radiology centers located in Central and Western Regions of Ghana were reviewed. Records of patients who presented with headache as principal complaint between January 2017 and December 2020 were reviewed. A total of 477 records of patients with headache were identified, retrieved and evaluated. A Chi-square test and Fisher exact test were used to compare the CECT and NCECT groups. Binary logistic regression analysis was computed to assess association between CECT and each CT findings. Statistical significance was considered at *p* < 0.05 with a 95% confidence interval.

**Results:**

A significant proportion of the patients was females (51.8% in CECT and 60% in NCECT). The NCECT group (40.06 ± 14.76 years) was relatively older than the CECT group (38.43 ± 17.64 years). There was a significant difference between the CECT and NCECT in terms of age (*p*=0.002) and facility CT was performed (*p* < 0.0001). The rate of abnormalities was higher in CECT (43.5%, 166/382) compared NCECT (37.9%, 36/95). There was no significant association between CT head findings and contrast enhancement.

**Conclusion:**

CECT examination accounted for 5.6% increase in the detection of head abnormalities. Efforts required to establish local standard operation procedures (SOPs) for contrast medium use especially in CT head examinations. Further studies to improve the knowledge of agents, mechanism of action, and safety of contrast media used among practitioners in Ghana is recommended.

## 1. Background

Contrast-enhanced material (CEM) has been used in neuroimaging since computed tomography (CT) was introduced. [1] CEM in the central nervous system (CNS) is a combination of two primary processes: intravascular enhancement and extravascular enhancement. [1–5] Whilst adapting CEM in diagnostic procedure, the referring physician and the radiologist should preliminarily: assess patient risk versus potential benefit of the contrast-assisted examination; consider imaging alternatives that would provide the same or better diagnostic information; and assure patient of a valid clinical indication for each contrast medium (CM) administration. [4, 6] Preparation for prompt treatment of CM reactions must include preparation for the entire spectrum of potential adverse events and include prearranged response planning with availability of appropriately trained personnel, equipment, and medications. [4].

Headache disorders are one of the most common disorders of the nervous system. [7] Headache is one of the principal complaints in both routine and emergency medical practice, and may primarily be caused by migraine, tension-type headache, and cluster headache. [7–9] The estimated prevalence in adults population of current headache disorder (symptomatic at least once within the last year) is about 50%. [7] About 50% to 70% of adults aged 18–65 years in the world have experienced headache in the last year and, among those individuals, 30% or more have reported migraine. [7] Apart from these common causes, there are multiple other causes such as trauma, vascular disorders, infections, tumors, and substance abuse. Some forms of headache like ophthalmoplegic migraine have a typical clinical presentation and imaging which may or may not be informative, while in others like neoplasms imaging offers an early diagnosis and a chance for potential treatment. [10].

Contrast CT scanning is increasingly used both in research and in clinical medicine worldwide, and the quality of images obtained with newer helical scanners is continually being improved. [8–11] CT, by virtue of its ubiquity, ease of use, speed, and lower financial footprint, is usually the first investigation to be asked for in cases of headache. [10] The use of contrast enhancement has improved diagnosis and treatment worldwide. [1, 5, 6, 12–16] However, not much has been reported from developing countries regarding these innovations of CECT and headache investigations.

In Ghana, CECT is widely used to diagnose and manage various complaints including headaches as it facilitates a comprehensive diagnosis and permits timely and targeted intervention. [17] In this study, we conducted a multicenter retrospective analysis to compare the findings of CECT to NCECT head examinations among patients presented with headache. Specifically, this study; described patients characteristic of CECT and NCECT head examinations, compared headache diagnosis between CECT and NCECT patients' groups, and assessed the association of contrast enhancement and head CT findings among patients with headache.

## 2. Materials and Methods

This was a multicenter retrospective analysis of four years' CT scan of the head records at two radiology centers located in Central and Western Regions of Ghana. Using a nonrandom purposive sampling method to select examinations, this analysis covered patients who presented with headache as principal complaint at Cape Coast Teaching Hospital (CCTH) Imaging department in Cape Coast and RAAJ Specialist Scan center in Takoradi between January 2017 and December 2020. The study protocol was approved by the Cape Coast Teaching Hospital Ethical Review Committee (ERC) (Ref: CCTHERC/EC/2022/059). However, the ERC waives consent for the use of secondary data.

Records of all patients aged one year or above, referred to the two radiology centers for a head CT scan with headache as principal indication, from any mechanism were included. The patient was excluded if the record had incomplete or missing relevant data.

**Figure 1:**
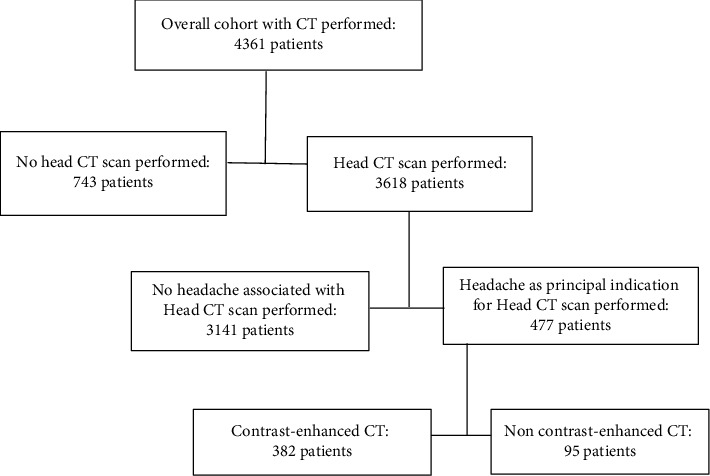
Flowchart of performed head CT scans in contrast medium in patients with any type of headache.

The database captured 3618 head CT examinations within the period, however, 477 formed the sample for this study. These were patients with headache of any type who were identified, retrieved from the database, and evaluated (Figure 1). Patients with abnormal head CT findings were classified into three groups: Brain parenchymal lesions only as Group A, nonbrain parenchymal lesions only as Group B, and those with both lesions as Group C. A checklist was developed as a guide for data extraction. The checklist captured each patient's unique identification (ID), date of request (day/month/year), age, sex, headache sub-types, whether CT scan was performed with or without contrast enhancement, documented adverse effect of contrast medium, and CT scan findings. Data were entered directly into a data extraction template on Microsoft Excel version 2020. We observed data confidentiality and security by using unique ID numbers instead of patient names and a password-protected laptop, respectively. Only investigators had access to the data.

### 2.1. Statistical Analysis

Data management and statistical analysis were performed using Statistical Package for Social Sciences (SPSS) software version 24. Descriptive Statistics such as frequencies and percentages were used to estimate the proportion of patients in age groups, sex, findings, and diagnosis. A Chi-square test and Fisher exact test (as appropriate) were used to compare the CECT and NCECT groups in terms of age, gender, facility, and headache sub-types. Binary logistic regression analysis was computed to assess association between CECT and each CT findings. Statistical significance was considered at *p* < 0.05 with a 95% confidence interval.

## 3. Results

### 3.1. Patients' Characteristics

A total of 477 patients with headache were reviewed between 2017 and 2020. The NCECT group (40.06 ± 14.76 years) was relatively older than the CECT group (38.43 ± 17.64 years). A significant proportion of the patients were females (51.8% in the CECT group and 60% in the NCECT group). There was a significant difference between the CECT and NCECT groups in terms of age (*p*=0.002) and facility (*p* < 0.0001) (Table 1).

### 3.2. Rate of Abnormal CT Head Findings

**Figure 2:**
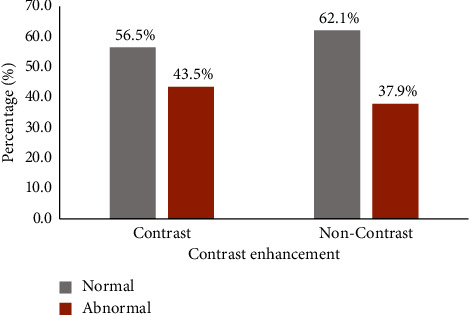
Rate of abnormal head CT findings among patients presenting with headache, 2017–2020.

The overall rate of abnormal CT findings was 42.35% (*n* = 202/477). The rate was higher in CECT (43.5%, 166/382) compared to NCECT groups (37.9%, 36/95) (Figure 2).

The comparison of abnormal diagnosis between CECT and NCECT patient groups is shown in Table 2. Generally, patients with abnormal CT diagnosis were older than the normal group. Males (51.6%) were likely to have abnormal diagnosis in the CECT group as compared to females (40.4%) in the NCECT group. Abnormal CECT head diagnosis was significantly associated with age groups (*p*=0.012) and gender of patient (*p*=0.002) (Table 2).

### 3.3. Classification of Head CT Findings in Contrast CT and Noncontrast CT

**Figure 3:**
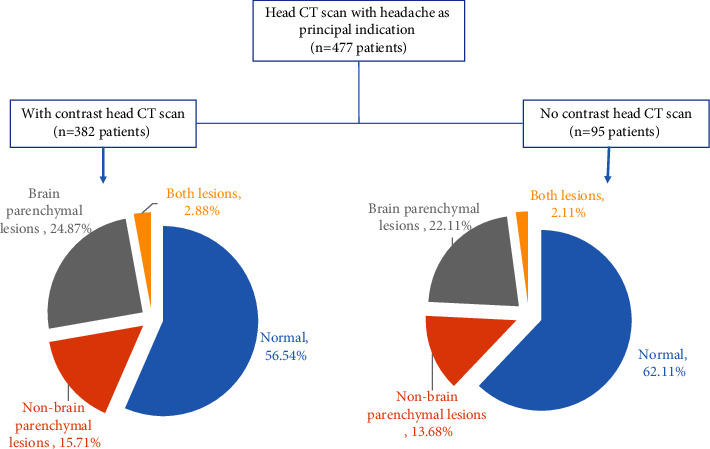
Distribution of patients with headache by classes of CT findings, 2017–2020.

Of 202 patients with CT abnormalities, 36.1% (*n* = 73), 57.4% (116), and 6.4% (13) were classified into brain parenchymal lesions, nonbrain parenchymal lesions, and both lesions. As shown in Figure 3, CECT examination accounted for 5.6% (43.5% cases with lesions with contrast verses 37.9% without contrast) increase in the detection of head abnormalities.

### 3.4. Head CT Findings

Table 3 illustrates head CT findings and contrast enhancement among patients with headache. In CECT groups, nearly 59%, 24.1%, and 12.7% of the patients had sinusitis, tumor/metastasis/cyst, and hemorrhage, respectively. Similarly, sinusitis and tumor/metastasis/cyst contributed 61.1% and 30.6% but fewer cases (8.3%) of hemorrhage was seen in the NCECT group. There was no significant association between head CT findings and presence of a contrast (Table 3).

### 3.5. Comparing the Noncontrast Phase of the CECT Group with the NCECT Group

Contrast-enhanced CT scan protocol involves the initial noncontrast phase followed by the contrast phase. All 382 patients in the CECT group had noncontrast CT prior to the administration of the contrast medium.

### 3.6. Sensitivity Analysis on the Contrast-enhanced CT Group

Further review and analysis of 382 patients in the CECT group showed that only 43 (11.3%) of patients who underwent contrast administration were necessary, without the contrast medium, the radiologist could still make the same impression made with the administration of contrast medium. This also represented 25.9% (43/166) of patients who had abnormal CT diagnosis with the contrast medium. It was evident from the analysis that the contrast medium was not necessary in the diagnosis of normal CT. The use of the contrast medium was much necessary in patients suspected with hydrocephalus/edema (58.3%), tumor (48.9%), meningitis (50%), and pathologic bone-diseases (50%) (Table 4).

### 3.7. Adverse Contrast Medium Reaction

There were no recorded adverse reactions from the contrast medium administration from both centers.

## 4. Discussion

The use of contrast enhancement has improved diagnosis worldwide. [1, 5, 6, 12–16] In Ghana, CECT is widely used to diagnose and manage various complaints including headaches as it facilitates a comprehensive diagnosis and permits timely and targeted intervention. [17] Radiographic examination of the head is an essential part of management of patients with various degrees of headache. [18–21] In this study, we conducted a multicenter retrospective analysis of CECT head examinations among patients presented with headache.

Consistent with previous studies, [8, 10, 17, 22] significant proportions of patients from both groups were aged between 20 and 50 years. This age group is the most active and productive group of our society and is more likely to be exposed to both occupational and social risks. [17] The age pattern is similar to demographic distribution in Ghana. [23] This study found that abnormal diagnosis was significantly associated with age groups and gender under CECT. Although we cannot directly explain the disparity between the CECT and NCECT group, the improved accuracy with contrast enhancement might have favored the distribution of cases by demography as observed in this study. This is inconsistent with the findings of Rai et al. [8] who reported no significant association between demographic factors (age and sex) and head CT findings.

The use of CECT accounted for 5.6% increase in the detection of CT head lesions. This finding affirms the evidence from literature that supports the effects of contrast enhancement on medical imaging. [1, 5, 6, 12–16, 24] However, we found no significant difference in the patterns of lesions detected between the CECT and NCECT groups. Extra-cranial lesions were detected in more than half of patients compared to intracranial lesions. [8, 17, 19, 25–27] The findings of the present study corroborate with Rai et al. [8] who reported a similar pattern of extra-cranial lesions and intracranial lesions. There was no statistical difference in the proportion of patients diagnosed with sinusitis and brain neoplasm (tumor, metastasis, and cyst) when CT was contrast enhanced. However, much lower proportion of hemorrhage was seen in NCECT. The high incidence of sinusitis is consistent with previous studies which significantly linked headache to different sinusitis. [28–30] Aydemir et al. [28] reported that headache is significantly associated with the mean maxillary, frontal, and sphenoid sinuses volumes, and the total sinus volumes (sum of maxillary, frontal, and sphenoid sinuses) of the patients. [28].

The contrast medium (CM) injected prior to CT scanning could enhance the accuracy in detection and interpretation of CT impressions. [6, 13, 16] Iohexol (Omnipaque), a low osmolar contrast medium (LOCM) was used at both centers. Low osmolality contrast media (LOCM) are favored for intravascular and intrathecal delivery since their osmolality is less than three times that of human serum. These are nonionic monomers made up of tri-iodinated benzene rings with different side chains containing polar alcohol (-OH) groups, which make them water-soluble. [31] As evident in the current study, facts from literature also validate the essence of CM in medical practice and imaging studies. [1, 5, 6, 13, 16, 24, 32] Several agents have been identified to enhance accurate diagnoses whether intravascular or extravascular CM. [2, 4, 12, 14, 24, 32] The use of intravascular contrast in radiology continues to increase compared to extravascular contrast agents. [2, 4, 33] The universally used agents, iodinated and gadolinium-based contrast media, are nearly always safe and effective when administered appropriately. [34] Recent radiologic studies and reports have confirmed that a major life-threatening contrast reaction is rare. [2, 4, 14, 34, 35] However, adverse side effects following the administration of CM vary from minor physiological disturbances to rare severe life-threatening situations. Therefore, the potential risks of intravascular administration of contrast must be weighed against the potential benefits. [2].

Local standard operation procedures (SOPs) for CM used in various diagnostic settings are lacking. This could further impact the benefits and safety of CM in radiography since the radiology practices are not aligned to known standards in Ghanaian context. Adaptation of foreign SOPs on CM use in medical imaging (such as the Royal College of Radiologists (RCR), Standards for intravascular contrast administration to adult patients and American College of Radiology (ACR) Manual on Contrast Media) [2, 4] is important but the Ghana Association of Radiologist must develop locally suitable SOPs that ensure standardization while serving as reference point for CM use in our context. In this document, three important issues must be well-covered; first, the indications necessitating the use of contrast medium after noncontrasting CT. Secondly, in any diagnostic procedure, the referring clinician and radiologist should consider the risk-to-benefit profile of the proposed contrast–enhanced examination and potential imaging alternatives that would provide the same or better diagnostic information and confirm a valid clinical indication. [2, 4] Thirdly, the preparation for timely treatment of CM reactions must include preparation for the entire spectrum of potential adverse events and include prearranged response planning with availability of appropriately trained personnel, equipment, and medications. [2–4, 34, 36].

### 4.1. Limitations of the Study

Foremost, this study could not classify the headache into the universally known sub-types (migraine, tension-type, and trigeminal autonomic cephalalgias). [7, 18] due to the lack of adequate patient history documentation on request by the clinicians. Moreover, this analysis was conducted with two-center data, the findings may not be generalizable to other centers in Ghana. As population characteristics may differ between medical centers, various prior probabilities may lead to different yields of CT head findings under CM. Despite these limitations, evidence from this study confirms the efficacy of contrast-enhanced CT in diagnosing abnormalities. It has improved knowledge and adds to existing data on the significance of CM in the Ghanaian context. Also, the gap (lack of local SOPs) has been identified to inform stakeholders' discussion and resolution to improve practice and patients' safety.

## 5. Conclusion

Contrast-enhanced CT of the head is a common practice and provides better diagnosis in patients with headache disorders. The use of CECT accounted for 5.6% increase in the detection of head lesions. Sinusitis, brain neoplasm, and hemorrhage were the common lesions from head CT scan under the contrast medium. There was statistical association between CECT head diagnosis and age and gender. A significant majority of the patients received the contrast medium but there was no significant association between head CT findings and CECT. Efforts are required to establish local SOPs for contrast medium use especially in CT head examinations. Further studies to improve the knowledge of agents, mechanism of action, and safety of contrast media used among practitioners in Ghana is recommended.

## Figures and Tables

**Table 1:** Patients characteristic of contrast CT and noncontrast CT head examinations, 2017–2020.

Variables	Category	Contrast enhancement		*P*-value
		Contrast, *n* = 382 (%)	Noncontrast, *N* = 95 (%)	
Age (years)	Mean (±SD)	38.43 (±17.64)	40.06 (±14.76)	0.002^*α*^
	1–10	10 (2.6)	2 (2.1)	
	11–20	36 (9.4)	1 (1.1)	
	21–30	42 (11.0)	10 (10.5)	
	31–40	75 (19.6)	12 (12.6)	
	41–50	46 (12.0)	10 (10.5)	
	51–60	31 (8.1)	8 (8.4)	
	61–70	13 (3.4)	4 (4.2)	
	>70	18 (4.7)	0 (0.0)	
	Unspecified	11 (29.1)	48 (50.5)	

Gender	Male	184 (48.2)	38 (40.0)	0.153^*β*^
	Female	198 (51.8)	57 (60.0)	

Facility	CCTH	194 (50.8)	3 (3.2)	<0.0001^*α*^
	RAAJ	188 (49.2)	92 (96.8)	

Type of headache	Acute/severe	25 (6.5)	5 (5.3)	0.572^*β*^
	Chronic	28 (7.3)	6 (6.3)	
	Traumatic	10 (2.6)	5 (5.3)	
	Unspecified	319 (83.5)	79 (83.2)	
	Category	Contrast enhancement	*P*-value	

CCTH: Cape Coast Teaching Hospital; RAAJ: RAAJ Specialist Scan center; *β* Chi-square test of association; *α* Fischer exact test of association.

**Table 2:** Comparison of contrast CT and noncontrast CT head diagnosis of patients presenting with headache, 2017–2020.

Category	Contrast		*P*-value	Noncontrast		*P*-value
	Abnormal, *n* = 166 (%)	Normal, *N* = 216 (%)		Abnormal, *n* = 36 (%)	Normal, *N* = 59 (%)	
*Age (years)*						
Mean (±SD)	42.79 (±19.88)	35.49 (±15.33)	0.012^*α*^	44.22 (±12.79)	37.48(±15.51)	0.598^*α*^
1–10	4 (40.0)	6 (60.0)		0 (0.0)	2 (100.0)	
11–20	10 (27.8)	26 (7.2)		0 (0.0)	1 (100.0)	
21–30	16 (38.1)	26 (61.9)		3 (30.0)	7 (70.0)	
31–40	25 (33.3)	50 (66.7)		6 (50.0)	6 (50.0)	
41–50	16 (34.8)	30 (65.2)		3 (30.0)	7 (70.00)	
51–60	19 (61.3)	12 (38.7)		3 (37.5)	5 (62.5)	
61–70	6 (46.2)	7 (53.9)		3 (75.0)	1 (25.0)	
>70	13 (72.2)	5 (27.8)		0 (0.0)	0 (0.0)	

*Gender*						
Male	95 (51.6)	89 (48.4)	0.002^*β*^	13 (34.2)	25 (65.8)	0.546^*β*^
Female	71 (35.9)	127 (64.1)		23 (40.4)	34 (59.7)	

*Facility*						
CCTH	75 (38.9)	119 (61.3)	0.055^*β*^	2 (66.7)	1 (33.3)	0.297^*α*^
RAAJ	91 (48.4)	97 (51.6)		34 (36.9)	58 (63.0)	

*Type of headache*						
Acute/severe	13 (52.0)	12 (48.0)	0.799^*β*^	2 (40.0)	3 (60.0)	0.210^*α*^
Chronic	12 (42.9)	16 (57.1)		3 (50.0)	3 (50.0)	
Traumatic	5 (50.0)	5 (50.0)		4 (80.0	1 (20.0)	
Unspecified	136 (42.6)	183 (57.4)		27 (34.2)	52 (65.8)	

CCTH: Cape Coast Teaching Hospital; RAAJ: RAAJ Specialist Scan Center; *β* Chi-square test of association; *α* Fischer exact test of association.

**Table 3:** Head CT findings and contrast enhancement among patients with headache, 2017–2020.

CT findings	With contrast		Without contrast		Logistics regression	
	Number (*n*)	^#^Percentage (%)	Number (*n* = 36)	^#^Percentage (%)	OR (95% CI)	*P*-value
Sinusitis	98	59.0	22	61.1	0.92 (0.44–1.92)	0.818
Hydrocephalus/edema	12	7.23	3	8.3	0.86 (0.23–3.21)	0.819
Hemorrhage	21	12.7	3	8.3	1.59 (0.45–5.66)	0.471
Meningitis	5	3.0	0	0.0	—	—
Tumor/metastasis/cyst	40	24.1	11	30.6	0.72 (0.33–1.60)	0.420
Infarct	11	6.6	3	8.3	0.78 (0.21–2.95)	0.715
Brain atrophy	8	4.8	0	0.0	—	—
Bone-related pathology	3	1.8	1	2.8	0.64 (0.07–6.38)	0.707
Ischemic small vessel	5	3.0	3	8.3	0.34 (0.08–1.50)	0.155
Calcifications	3	1.8	1	2.8	0.64 (0.07–6.38)	0.707
^o^Other CT findings	7	4.2	1	2.8	1.54 (0.83–12.93)	0.690

^Φ^ Other CT findings include mastoiditis (3), thrombosis (1) diffuse deep white matter (1), enlarged tonsils (1), and herniation (1); ^#^ the total is more than 100%, one patient could be diagnosed of multiple pathologies.

**Table 4:** Sensitivity analysis of the contrast-enhanced CT group of patients with headache, 2017–2020.

Variables	Frequency	Necessity of contrast medium	
		Necessary, *n* = 43 (%)	Unnecessary, *n* = 339 (%)
CT diagnosis			
Abnormal	166	43 (25.9)	123 (74.1)
Normal	216	0 (0.0)	216 (100.0)
CT findings			
Sinusitis	110	16 (14.5)	94 (85.5)
Hydrocephalus/edema	12	7 (58.3)	5 (41.7)
Hemorrhage	8	2 (25.0)	6 (75.0)
Meningitis	4	2 (50.0)	2 (50.0)
Tumor/metastasis/cyst	45	22 (48.9)	23 (51.1)
Infarct	6	1 (16.7)	5 (83.3)
Brain atrophy	8	2 (25.0)	6 (75.0)
Bone-related pathology	4	2 (50.0)	2 (50.0)
Ischemic small vessel	7	0 (0.0)	7 (100.0)
Calcifications	4	1 (25.0)	3 (75.0)

## Data Availability

The data used to support the findings of this study are included within the article.
